# Voice-Hearing and Personification: Characterizing Social Qualities of Auditory Verbal Hallucinations in Early Psychosis

**DOI:** 10.1093/schbul/sbaa095

**Published:** 2020-07-16

**Authors:** Ben Alderson-Day, Angela Woods, Peter Moseley, Stephanie Common, Felicity Deamer, Guy Dodgson, Charles Fernyhough

**Affiliations:** 1 Department of Psychology, Durham University, Science Laboratories, Durham, UK; 2 Department of English Studies, Durham University, Durham, UK; 3 Department of Psychology, Northumbria University, Northumberland Building, Newcastle upon Tyne, UK; 4 Cumbria, Northumberland, Tyne, and Wear NHS Foundation Trust, St. Nicholas Hospital, Newcastle upon Tyne, UK; 5 Tees, Esk, and Wear Valley NHS Foundation Trust, West Park Hospital, Darlington, UK; 6 Institute of Forensic Linguistics, Aston University, Birmingham, UK

**Keywords:** schizophrenia, cognitive behavioral therapy, early intervention, social cognition, psychopathology

## Abstract

Recent therapeutic approaches to auditory verbal hallucinations (AVH) exploit the person-like qualities of voices. Little is known, however, about how, why, and when AVH become personified. We aimed to investigate personification in individuals’ early voice-hearing experiences. We invited Early Intervention in Psychosis (EIP) service users aged 16–65 to participate in a semistructured interview on AVH phenomenology. Forty voice-hearers (*M* = 114.13 days in EIP) were recruited through 2 National Health Service trusts in northern England. We used content and thematic analysis to code the interviews and then statistically examined key associations with personification. Some participants had heard voices intermittently for multiple years prior to clinical involvement (*M* = 74.38 months), although distressing voice onset was typically more recent (median = 12 months). Participants reported a range of negative emotions (predominantly fear, 60%, 24/40, and anxiety, 62.5%, 26/40), visual hallucinations (75%, 30/40), bodily states (65%, 25/40), and “felt presences” (52.5%, 21/40) in relation to voices. Complex personification, reported by a sizeable minority (16/40, 40%), was associated with experiencing voices as conversational (odds ratio [OR] = 2.56) and companionable (OR = 3.19) but not as commanding or trauma-related. Neither age of AVH onset nor time since onset related to personification. Our findings highlight significant personification of AVH even at first clinical presentation. Personified voices appear to be distinguished less by their intrinsic properties, commanding qualities, or connection with trauma than by their affordances for conversation and companionship.

## Introduction

Auditory verbal hallucinations (AVH)—hearing voices that others cannot hear—are a prominent feature of psychotic disorders. Not all warrant psychiatric care, but AVH are often distressing, debilitating, and persistent, despite treatment with antipsychotic medication and cognitive behavioral therapy (CBT) for psychosis.^1^

Recent therapeutic approaches to AVH have gained attention by encouraging voice-hearers to talk to their voices. This may involve “empty chair” work, role-play, or dialoguing with a computer simulation of a distressing voice.^2–4^ Such techniques exploit person-like qualities of AVH and treat them as entities that can be conversed with meaningfully. Talking to voices has not always been encouraged for various reasons, including fear of reinforcing beliefs about the reality of the experience. However, promising results in long-term voice-hearers suggest that dialogue may be considerably beneficial for some.^5^ To understand the broader suitability of such methods, we first need to ask whether, why, and when AVH become personified.

Influential theories posit voice personification as a secondary response to a primary hallucinatory experience, which may be elaborated over time.^6,7^ Much focus has been on *who* the voices represent and whether voice identity, real or unreal,^8,9^ reflects delusional thinking.^7^ Prominent cognitive approaches to AVH have emphasized beliefs concerning voice power and omnipotence, orienting therapy toward challenging commands from malevolent voices.^10,11^

Personification, though, consists of more than identity and power; something being “like a person” may also involve ascribing animacy, agency, physical features, intentions, or linguistic complexity.^12–15^ Recent phenomenological research has emphasized the multimodal and embodied nature of AVH: eg, voices may be described as having presence or “appearing” in more than 1 modality.^16^ Similarly, voices may be experienced with an emotional depth that goes beyond fears of omnipotence and malevolence.^17–19^ These considerations imply that social and agent-like properties of AVH are primary to the experience rather than secondary interpretations.^20^ This aligns with trauma-informed and Hearing Voices Movement approaches, where voices are often understood as reflecting past relationships and interpersonal trauma.^17,21,22^

Understanding personification in AVH requires a clear account of what voices are like when they start.^13^ Few studies, however, have focused on early AVH phenomenology, with researchers relying instead on retrospective accounts from long-term voice-hearers of how their voices began.^17,20^ Some qualitative studies have collected longitudinal data on voices,^23–25^ but they have not closely tracked early phenomenology or personification, instead focusing on beliefs about voices. Exploring voices’ initial presentation—or as early as possible—is key to understanding their potential person-like qualities. Moreover, it allows for a closer examination of the psychological, biographical, and social context in which personification emerges.

Here, we present findings exploring AVH personification in a group of new Early Intervention in Psychosis (EIP) users. Although many people hear voices *before* using services, recruitment of such individuals can be highly challenging; we chose EIP to provide a pragmatic snapshot of early clinical presentation in a large, regional UK service. Within this context, assessment may be influenced by a range of clinical concerns (such as risk or cognitive appraisal) and this is reflected in the focus of many standard AVH interviews.^26,27^ For a complex topic such as personification, it is important to look beyond existing clinical constructs and draw upon multiple kinds of expertise, both from experts-by-experience and across academic disciplines.^28^ Our previous study^16^ used an online phenomenological survey with the input of multiple disciplines and lived experience researchers to explore unexamined properties of voice-hearing, identifying high rates of characterful (69%) and embodied (66%) experiences of voices. Here, we used a similar approach to explore AVH characteristics in more depth using a semistructured interview that focused on early presentation and person-like qualities of voices (characteristics not typically emphasized in prior surveys^9,27^). We used a mixed-methods approach to characterize the degree of personification evident in the sample alongside other phenomenological and clinical characteristics.^8,29^ We identified common associations with “person-like” voices and examined whether personification reflects trauma and commanding voices—as suggested previously^11,21^—or is in itself an important, independent dimension of AVH.

## Method

### Participants

Users of 2 EIP services in northern England aged 16–65 were invited to take part if they heard voices at least once a week for a month, had normal or corrected-to-normal vision, had been in EIP under 9 months, and were fluent English speakers. Exclusion criteria were the presence of a suspected duration of untreated psychosis over 5 years (ie, not just voices but other psychotic experiences and/or deterioration of function), any neurological diagnoses, or having a hearing impairment that required the use of hearing aids. Participant information sheets did not define AVH characteristics in advance but referred to “hearing voices that others cannot hear.” Recruitment was open from September 2017 to April 2019 and was conducted primarily via case-list review. This cohort is being followed up using the same protocol at 12 and 24 months following entry into the study. A pragmatic sample of 40 participants was recruited to enable in-depth qualitative interviewing and analysis, exploratory quantitative analysis, and longitudinal follow-up. All procedures were approved by a local National Health Service (NHS) Research Ethics Committee.

## Materials

### The Hearing the Voice Phenomenology Interview

Participants took part in a semistructured interview with 1 of 2 interviewers trained in clinical, phenomenological, and qualitative health interviewing. Following our previous survey,^16^ 8 open-ended questions about AVH were used to elicit discussion, followed by prompts allowing for elaboration. The interview was developed by an interdisciplinary team (including psychologists, philosophers, linguists, theologians, literary and medical humanities scholars) in consultation with experts-by-experience and with service-user input into its design and acceptability (see supplementary material). Bracketing assumptions about voice-hearing experiences, questions progressed from general (“Please could you describe the voice or voice-like experiences you have been having?”) to specific, being careful not to introduce suggestions of character or presence until participants’ own descriptions and interpretations were firmly established in the interview. Sessions typically took 1 h (range 24–105 min).

### Psychotic Symptoms Rating Scale

The Psychotic Symptoms Rating Scale (PSYRATS) is a common tool for assessing the severity of hallucinations and delusions in people with psychosis.^27^ Ratings are made by the interviewer ranging from 0 to 4 (absent to most severe). The PSYRATS was used to examine how phenomenological properties of voices related to standardized ratings of severity and distress. As part of a wider study, subsets of participants also completed self-report measures of delusional thinking, inner speech, hallucination proneness, loneliness, and functioning, plus a cognitive assessment and magnetic resonance imaging scan as part of separate sessions. These data will be reported elsewhere.

After providing written consent to take part, participants completed the phenomenology interview and PSYRATS with the interviewer. Sessions took place in participants’ homes, NHS settings, or a university room. All interviews were recorded and then professionally transcribed for analysis.

### Analysis

Interview data were analyzed using a mixture of qualitative and quantitative methods. Content analysis and inductive thematic analysis^30^ were used to derive a coding frame that permitted direct comparison with prior phenomenological surveys of AVH,^8,9,16^ while also allowing for a nuanced analysis of the specific qualities of the data collected. This was developed iteratively by an interdisciplinary team (2 psychologists and a medical humanities scholar) who met after each interview to discuss new codes, co-code 7 interviews, discuss and resolve disagreements, and then code the remaining interviews independently. Interrater reliability was satisfactory using the 3-way rating permitted by Krippendorff’s alpha (α = .70). Two recent service users with lived experience of voices also read and discussed the anonymized interview transcripts with the research team during the analysis and writing-up period, which primarily informed general interpretations of the main findings.

Quantitative analysis was used to examine common associations among codes of interest and to compare participants with and without key codes on continuous outcomes (such as levels of distress). Due to the exploratory nature of the research question, a descriptive approach was deployed using log odds ratios (lgORs) and effect sizes (Cohen’s *d*) to indicate the strengths of association. All analyses were conducted in R using the *jmv* package. For parsimony, we have only focused our discussion on odds ratios (ORs) with confidence intervals not crossing 0. Unless included in figures or tables, all other ORs and confidence intervals are reported in supplementary material. R code and quantitative data are available at https://osf.io/arj86/.

## Result

Forty participants took part (Age *M* [SD] = 28.70 [9.96] years). The average amount of time in EIP was just under 4 months (*M* [SD] = 114.13 [64.77] days). Thirty-two (80%) were currently taking antipsychotic medication, while 42.5% had had access to some form of psychological therapy (CBT in 11 cases). At the time of assessment, 45% did not have any clinical diagnosis, consistent with the fact that distressing symptom presence rather than the fulfillment of diagnostic criteria is an entry requirement for EIP. 42.5% had a psychotic disorder diagnosis (substance-induced psychosis 2.5%, schizophrenia 5%, depression with psychotic features 10%, and unspecified psychosis 25%), while the remaining 12.5% had a nonpsychotic diagnosis (5% emotionally unstable personality disorder, 5% post-traumatic stress disorder, and 2.5% delirium). Reflecting regional norms, all but 1 participant was of white British ethnicity (1/40 British Asian).

Despite their short time in services, participants had been hearing voices for 74.38 months (SD = 81.24, range 1–329). Mean age of voice onset was 22.68 years (SD = 13.45; this estimate excludes 1 participant who could only say that it first happened “a very long time ago”). In 9 cases, onset did not coincide with need-for-care (age *M* [SD] = 11.11 [7.97] years), with distressing voices only appearing many years later (*M* [SD] = 20.67 [9.02]). For those whose first experience was distressing (*n* = 30), age of onset was often higher (*M* [SD] = 26.15 [12.87] years, *d* = 1.26). Here, participants had typically been hearing negative voices of some kind intermittently for less than 4 years (*M* [SD] = 43.93 [56.96] months, median = 12 months). Earlier onsets in life were often described with more benign voice content and interpretation than later onsets (see box 1a).

**Box 1a. When Voices Start**

I can hear her since I was like six.... Actually see her from about sixteen I’d say.… she was just copying, taking the mick out of me a little bit when I was little…. But then like when I was little, I saw it as imaginary friend type of thing, I just put it down to that…. And then when I got to about thirteen, fourteen, she got on about me weight a lot. And when I was sixteen, it got to about her hurting me because I was trying to lose weight so much. [Orla, age 19]

They started in … the voices that actually talk to me, I’m overhearing conversations. And they started the beginning of November last year, when I believed I was witnessing either a… a sex ring or a drug ring outside… and initially it was only a couple of nights a week, gradually it became more and more, and what I believed I was hearing was… turned into an undercover operation, involving police officers who were part of, who had infiltrated the ring, and were part of this ring that was going on outside. 99% of the time it was voices that I heard, sometimes I heard footsteps, but it was all me witnessing things outside me house. [Jade, age 62]

Table 1 displays the sensory characteristics described by participants (see supplementary material for all code definitions). Most participants reported literal *auditory* voices, but 52.5% also reported voices that had thought-like qualities. Other senses featured prominently, including *visual* (75%) and *olfactory* (37.5%) hallucinations. Two-thirds of the sample reported *bodily* changes associated with AVH, while 52.5% reported *felt presences*, in which voices (or other entities) were experienced as being present without speaking. *Multimodal* voices, where more than 1 sensory experience was explicitly connected to the voice (eg, the voice could be seen or smelled, even if not simultaneously heard; see box 1b), were reported by 11 participants.

**Table 1.** Sensory quality, space, control, and change over time

Codes	*n*	%
Sensory qualities and modality		
Auditory	37	92.5
Thought like	21	52.5
Nonverbal	16	40
Visual hallucination	30	75
Visual imagery	2	5
Tactile hallucination	9	22.5
Olfactory hallucination	15	37.5
Gustatory hallucinations	0	0
Dissociative experiences	12	30
Bodily states	26	65
Felt presence	21	52.5
Multimodal voices	11	27.5
Spatiality		
Internal	25	62.5
External	32	80
Egocentric voices	28	70
Allocentric voices	15	37.5
“Boundary” voices	14	35
Control and change		
Nonvolitional occurrence	40	100
Volitional occurrence	1	2.5
Ability to influence voice	11	27.5
Change in influence	5	12.5
Change in frequency	28	70
Change in number or structure of voices over time	32	80

**Box 1b. Multimodal Voices**

It’s a couple of voices, like throughout the years it’s always been a couple of voices… but then I started seeing the person as well, and then I could… like physically I guess I could touch them and I could hear them because I could see them talking as well. [Will]

I see him…. It’s normally like in the doorway…. Or sometimes like in the seat next to us, kind of thing. And upstairs when I’m bed as well…. Yeah, yeah, the corner of me eye, I can see him.... [Ian]

It’s… pretty much every time when she talks to me outside [my head]… I can see her. Or like I could see her when she’s just whispering… she doesn’t have to be talking directly to me, but like I know she’s there…. And like you can physically see her as she’s speaking to us and her mouth’s moving… I can see her standing in the room… I could feel her presence… the way she moves is just like another person standing there. [Orla]

As well as coding for *internally* and *externally located* voices, we coded for voices that were positioned either in relation to the voice-hearer (*egocentric*; 70%), their environment, such as specific rooms or when outside (*allocentric*; 37.5%), and *boundary voices*, ie, voices that were predominantly experienced at thresholds, such as doors or walls (35%). Here, the location of voices was often described in terms of a struggle to establish who was speaking or what was happening beyond their immediate space.

All participants reported voices being associated with *negative emotions*—most notably, *anxiety* and *fear*—but 35% also reported *positive emotions* relating to their voices (see table 2). The majority (67.5%) reported voices that provided them with *commands* to act, ranging from mundane imperatives to clear instructions to harm (mostly self-directed). Other voices *commented* on day-to-day actions and thoughts (45%) or could be engaged in *conversation* (47.5%). Despite the overall negativity of many of the voices described, 32.5% reported their voices providing a sense of *companionship*.

**Table 2.** Affect, agency, and interpretation

Codes	*n*	%
Affect and content		
Voices elicit positive emotions	14	35
Voices elicit negative emotions	40	100
1. Anxiety associated	26	65
2. Depression associated	21	52.5
3. Fear associated	24	60
4. Paranoia associated	20	50
Simple linguistic structure	16	40
Directly address voice-hearer	33	82.5
Voices comment on voice-hearer	18	45
Voices converse with voice-hearer	19	47.5
Commanding voices	27	67.5
1. Voice-hearer follows commands	13	32.5
Abusive/violent voices	35	87.5
Positive/helpful voices	17	42.5
Companionship from voice	13	32.5
Voice knows more than voice-hearer	18	45
Agency and character		
Recurring voices	37	92.5
Voices recognizable from voice-hearer’s life	19	47.5
Change in character or personality of voices across time	7	17.5
Absent agency^a^	6	15
Agency without individuation^a^	18	45
Internally individualized agency^a^	30	75
Externally individualized agency^a^	20	50
Minimal personification^b^	24	60
Complex personification^b^	16	40
Archetypal features	17	42.5
Social context and interpretation		
Voices important to identity/sense of self	5	12.5
Positive impact on relationships	2	5
Negative impact on relationships	31	77.5
Self-stigma regarding voices	19	47.5
Suicidal thoughts or actions	20	50
Sleep disruption	25	62.5
Traumatic context around onset	26	65
Trauma interpretation	10	25
Biophysical interpretation	10	25
Stress interpretation	15	37.5
Idiosyncratic interpretation	13	32.5
Supernatural/spiritual interpretation	8	20
Family narrative	9	22.5

Almost all participants reported specifically *recurring* voices (92.5%), and a large proportion (47.5%) described voices that were *recognizable* as real people. Wilkinson and Bell^20^ propose 4 levels of AVH agency: using these criteria, most participants described voices that recurred over time, had a distinct character, but could not be related to a known person (*internally individuated agency*; 75%).

Finally, we categorized voice-hearers’ interpretations and the impact of voices on daily life and relationships. No single interpretation was predominant—with many participants holding more than one kind of explanation at the same time—although *stress* explanations (such as poor sleep, physical health, and problems at work/school) were the most common (37.5%). Understanding voices as a response to *trauma*—which may be thought to bring out more person-like approaches to voices^17^—was discussed by only 25% of the sample, despite trauma of various kinds being reported around voice onset for over half the sample (26/40).

### Associations With Personification

Based on our reading of the interviews, we coded voice personification in 2 ways. Often only 1 or 2 references were made to voices as persons, such as a name or a general manner (*minimal personification*), but sometimes multiple references to qualitatively different person-like properties were made (*complex personification*; see box 2 for a full code description). Personification was evident in all interviews, although the majority (24/40) described minimally personified voices.

**Box 2. Minimal and Complex Personification**

**Minimal:** The voice has few person-like qualities: is attributed to a person or described as being “like a person” but without further elaboration. Person-like characteristics tend to remain stable over time and follow a single theme (eg, the voice is “mean” or a “nasty man”).

It’s a female voice, as if she’s in the room with me… I wouldn’t say she’s nasty, she’s just really stern and to the point… it’s mainly to do with me being a bad mum and like the guilt of… it just drives me nuts…. [Dawn]

Mine are just some lad that just chatters on about crap constantly…. Different voices, yeah, and then, then it dwindled down to one… it’s hard to explain what somebody’s voice is like but you know… middle-aged… that’s about all, you know? Kinda, kinda like it’s coming through you know like some sort of voice thing…. They have some sort of accent, but I can’t really tell, it’s probably from round here, you know. [Yan]

**Complex:** The voice is described as having more than one kind of person-like quality: may include elaborate descriptions of intentional states (the voice wants/thinks/feels), agency (the voice will “make something happen”), or identity (the voice “comes” from somewhere or has a specific and idiosyncratic ontological status). Complexity is not a simple function of the frequency, quantity, or topic of speech but will typically involve a voice being attributed multiple, qualitatively different person-like qualities (eg, voice has an identity *and* multiple mental states), which may vary over time.

When I was a teenager, I used to go out when I needed to get out the house, from my parents, my family and so on, I would walk and talk to her, and she’d be the one that always held my hand, and ever since… she’ll be the one that’ll say, ‘you will stop now, calm down, it’s OK, I’ll keep you safe, let’s go home’, and she’ll be the one that I’ll s… I can sort of see… it’s a nice, gentle face, and she just sort of hovers there in… yeah, and next to me. And she’ll be the one that kind of manages to get me home. [Eric]

I’ve got two main voices that I hear, one of them’s a girl and one of them’s a boy, ehm, weird man… and the girl’s really quite nice…. Like she makes us feel really good.… She sounds quite young, like I’m going to say maybes… I don’t know, like a young girl kind of thing, maybes like… ten, eleven, and she’s like very child-like in like she’s like, ‘oh [name] you look wonderful today’ and like ‘life’s glorious, like let’s do this and let’s do that’.... I know that the little girl’s like proper bubbly, like she loves life like you know what I mean? She loves everything.… I dunno, she’s just like a typical kid you know what I mean, like where they see optimism in everything and like… yeah. [Kath]

Well, it’s like every single day I hear three different voices, there’s a female voice, she’s called Bex, she actually introduced herself to me, and then there’s an angry male voice, who’s like there all the time, I don’t know his name, but he’s there like constantly unless I’m asleep, he like, he’s like… a boy in a way but like he just, he’s a bully to everyone. And then the third one is very quiet and… just he doesn’t, like I don’t hear him like every day, it might be like once a week that I hear him.… Bex can be quiet, like she doesn’t talk all the time, but he’s, the angry male voice is like constant… with the very quiet male voice, who I don’t hear, like once a week I’ll hear him, he’s a surprise, but with Bex, it’s weird, I, like I can sense that she’s going to talk. Bex mainly talks to me… she’ll argue with the angry male voice, telling him to like shut up and that, but… at the same… she… I feel like she holds back a lot because she, she knows how like exhausting it can be, like… hearing that…. Bex will be happy and you can tell she’s happy, or you know I wouldn’t say she’s sad but you can tell when she’s not happy, but she won’t be sad…. [Xander]

N.b. Voice and voice-hearer names have been changed to preserve anonymity.

To explore the nature of person-like voices in the cohort, we then analyzed *complex personification* for its co-occurrence with other codes. Based on prior theory,^13,31,32^ various person-like characteristics could be expected to predominately associate with personification; eg, *multimodality, felt presence, voice knowledge* (voice possesses knowledge separate from the voice-hearer), *ability to influence* (voice changes in response to the voice-hearer, ie, flexible agency), *conversational* (voice can be conversed with), and *companionship* (voice provides company or support). Personified voices might also be expected to have a *recognizable identity* to *change in character* over time and to have no *simple linguistic structure*.

**Fig. 1.**
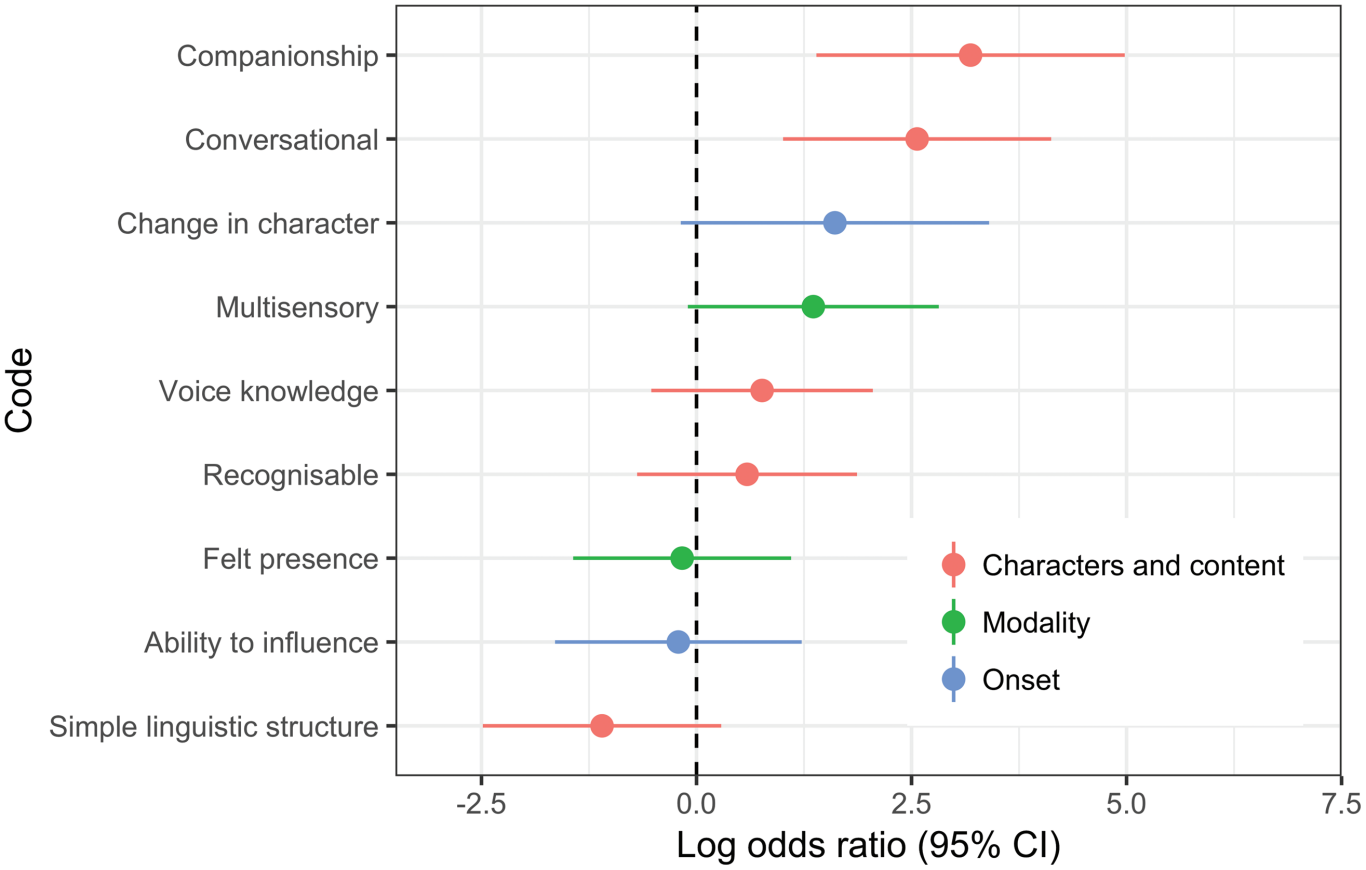
Specific person-like properties associated with complex personification of voices (rightward point estimates indicate greater association).

These predictions were only partially supported: positive associations with personification were evident for *companionship* (lgOR = 3.19) and *conversational* voices (lgOR = 2.56), but weaker associations were evident for other theoretically person-like properties (see figure 1). *Felt presence* showed the lowest association with personification among all sensory codes (lgOR = −0.17), with *visual hallucinations* scoring highest (lgOR = 2.20). Across all codes (see supplementary material), complex personification was most associated with voices being experienced as *positive-helpful* in character (lgOR = 3.89) and *eliciting positive emotions* (lgOR = 3.50), even while being predominantly negative.

Oddly, personification also coincided with voice-hearers reporting experiences that were *absent of agency* (lgOR = 2.35). Further inspection of these cases identified nonagentic experiences (random banging and recording-like voices) occurring alongside other highly agentic voices: eg, “Dan,” who hears over 5 voices, described a “computer-generated” voice generating “random stuff” (ie, *absent agency*), plus other voices that were capable of being “fake,” “manipulative,” “respectable,” and “trustworthy.” Supporting this, those with complex personification also tended to describe voices across more levels of agency^20^ (mean diff. = +0.67; *d* = 0.79) and in more modalities (mean diff. = +0.66, *d* = 0.59). To identify potential confounds, we also checked the association of complex personification with gender (lgOR = −2.43, with female participants [12/17] more likely to receive the code than males [4/23]), street drug use (lgOR = −1.89, with absent drug use associated with complex personification), and the presence of a diagnosis (no association evident, lgOR = 0.08).

Two codes with putative theoretical and causal links to voice personification are *commanding voices*^11^ and *presence of trauma* when voices began. Neither commanding voices (lgOR = 0.10) nor trauma (lgOR = −0.64) were associated with personification itself, and they were generally unrelated to person-like qualities of voices. Commanding voices were associated with *multimodality* (lgOR = 2.93)—with the latter fully coinciding with commanding experiences (11/11)—but were no more likely to occur with categories such as *companionship* (lgOR = 1.33), *conversational* voices (lgOR = 1.03), or *felt presence* (lgOR = −0.08). Instead, when compared to all codes, commanding voices were linked most strongly to *suicidality* (lgOR = 3.35), voices being *abusive/violent* (lgOR = 2.45), and *direct forms of address* (lgOR = 2.06; see supplementary material). Trauma around voice onset, conversely, was associated with *commanding* voices (lgOR = 1.72) and *stress-based* narratives (lgOR = 1.79), but associations with person-like qualities were generally low (eg, *conversational*; lgOR = −0.15).

Finally, we explored the relations between personification, age of voice onset, length of time hearing voices, and PSYRATS scores. No differences were evident (*d* = −0.12 to 0.05), suggesting that personification was unrelated to when voices started, how long they had been present, and overall symptom ratings (see supplementary material).

## Discussion

Are the person-like qualities of AVH present at their first clinical presentation or developed over time? Consistent with a broader “new look” at the phenomenology of psychosis,^9,15,16,28^ the data presented here highlight the complexity of AVH in EIP services. Almost all participants described multiple, recurring voices associated with negative emotions; however, a variety of auditory and thought-like voices, accompanying somatic, felt presence, and multimodal experiences, and positive emotions were also reported.

Personification is a concept that has not been systematically explored in research on voice-hearing, with prior work tending to focus on voice identity specifically, or the idea of relating to voices more generally.^8,20^ Most voices in the present study were “internally individuated” ^20^: ie, recognized as a recurrent voice but not attributed to an external agent. However, we investigated not just who voices represent but how they are experienced, identifying a significant subset of individuals for whom voices have multiple person-like qualities, including intentions, dispositions, and capacities for action. While this could have co-occurred with any number of phenomenological variables, voices with complex personification stood out as affording companionship and conversation. In other words, highly personified voices are distinguished less by their intrinsic properties (such as identity, linguistic complexity, modality, or presence) than by what they can do (afford engagement in dialogue) and their role in the life of the voice-hearer (as companions): they represent a pragmatic opportunity for the voice-hearer.^32^ They are also, curiously, reported alongside experiences distinctly *lacking* in agency. This could simply reflect a greater diversity of experiences in general for those with more complex voices but also suggests that personification may rely on contrast and comparison *across* voices, with the perception of personhood being a relative and comparative quality to assign. In this respect, AVH personification may be understood as the product, or reflection, of the different relational roles that voices can sometimes take up^33^.

Although commanding voices and the presence of trauma were both prominent in our sample—and of clear clinical import—neither seemed to drive personification. Indeed, our findings suggest that multimodality, rather than degree of personification, may have a greater role in the experience of commanding voices. In addition, voice personification was unrelated to overall PSYRATS scores for either hallucinations or delusions, suggesting that person-like voices do not necessarily reflect a greater severity of psychosis or delusional ideation.^7^

A quarter of our sample had been hearing voices for many years before entering EIP for the first time. Although these participants reported higher levels of current distress (see supplementary material), often their first experiences were not negative. By contrast, first voices were almost always distressing for participants with an adult onset. This accords with previous observations of early voice-hearing onsets in nonclinical samples, raising questions for the interaction of life stage and appraisal on AVH development.^34–36^ Perhaps surprisingly, neither age-of-onset nor time spent hearing voices related to the degree to which voices were personified. We cannot rule out that some voices will go on to develop person-like qualities or that elaboration over time may work differently for those with complex versus minimally personified voices, but it does suggest that personification does not straightforwardly reflect some secondary interpretation that grows over time.

If some voices simply *are,* from the outset, experiences that afford interaction, this has implications for the preponderance of new therapies that encourage such relations.^2,3,5^ An important limitation to consider here, though, is the possible role of gender and drug use: male participants and drug users were much less likely to experience strongly personified voices, which may confound our observed associations with companionship and conversational voices, and could influence therapy choices. Given that many in EIP services will be both male and using drugs, this could limit the relevance of relational therapies at early stages of psychosis (notwithstanding the evidence of extensive personification we identify). If the characterological resources are not there, there may not be “enough” to relate to or interact with for some voice-hearers.

More general limitations of the study are the reliance on self-report and the ability of participants to distinguish and remember their first experiences, which, in some cases, were many years prior to contact with EIP services. Despite our attempts to explore early voice-hearing, it should be acknowledged that voice onsets were highly varied—sometimes having occurred many years previously—increasing our reliance on long-term retrospective accounts. The focus on EIP users self-reporting voice-hearing also requires an individual to recognize, in some minimal sense, that their experiences are not part of a shared reality: we, therefore, cannot comment on personification in individuals who fully believe in the veridicality of their voices and do not recognize “voice-hearing” as a description that applies to their experience. Finally, the diagnostic heterogeneity of an EIP sample and lack of ethnic diversity regionally limits strong generalizations to other clinical groups and populations. For diagnosis, this may change over time (especially, considering that the sizeable minority of our participants had not received a psychiatric diagnosis), but closer examination of personification in its relation to diagnostic groupings is clearly required. As such, this work should be considered a starting point as we go about developing a more systematic understanding of voice personification.

The bracketing of presuppositions required in phenomenological investigation^28^—insofar as this is ever truly possible—demands an exploratory stance as we track this cohort longitudinally. Nevertheless, we can advance several tentative predictions. If the intensity and frequency of individuals’ AVH does recede over time and, through contact with EIP services, we might expect to see a related reduction in phenomenological complexity (particularly where related to sensory modality). By contrast, our data suggest neither a clear reduction nor elaboration of voice personification across time. While many voices show person-like qualities, we predict that complex personification will occur only where an emotional role or pragmatic function for a voice—such as companionship—is also present. These data—combined with other findings^25^—should allow us to answer the twin questions of whether there *is* always a speaker behind the voice and for which voice-hearers that might matter most in the longer term.

## Supplementary Material

sbaa095_suppl_Supplementary_Material

## Notes

**Author contributions:** The study was originally conceived by authors B.A.D., A.W., C.F. with P.M., G.D., and F.D. also contributing to study design. Data collection was conducted by B.A.D., P.M., S.C., G.D., and F.D., with analysis conducted by B.A.D., P.M., and A.W. All authors contributed to data interpretation and writing of the manuscript.
