# Supplementary material for: Voice-Hearing and Personification: Characterizing Social Qualities of Auditory Verbal Hallucinations in Early Psychosis
Source: Schizophr Bull. 2020 Jul 16;47(1):228–36. doi: 10.1093/schbul/sbaa095 (PMC7824995; doi:10.1093/schbul/sbaa095)
Supplement: sbaa095_suppl_Supplementary_Material [file sbaa095_suppl_supplementary_material.docx]

**Supplementary materials: “Voice-hearing and personification: Characterising social qualities of auditory verbal hallucinations in early psychosis”**

**Section 1. The Hearing the Voice Phenomenological Interview: background and example.**

A range of scales and measures currently exist for the assessment of auditory verbal hallucinations (AVH) in particular and psychotic experiences more generally, but none have focused on the phenomenology of AVH personification in detail. Those more focused on AVH are typically tailored towards establishing overall “severity” scores and rarely go beyond voice identity in their exploration of personification (e.g., Haddock et al., 1999). More phenomenologically detailed tools, such as the Examination of Anomalous Self Experience (Parnas et al., 2005), do not characterize AVH in depth, instead focusing on other psychotic experiences.

Our earlier phenomenology survey (Woods et al., 2015) was developed as part of an interdisciplinary research project (Hearing the Voice; Fernyhough, 2014; Woods et al., 2014; Woods and Fernyhough, 2014) and it successfully characterized a range of heterogeneous experiences that are not emphasized in work with existing AVH scales and surveys (such as embodied qualities of voices). Adapting our 2015 survey into an interview for the present study required input from the wider Hearing the Voice team - including scholars of philosophy, religious studies, literary studies, linguistics, psychology, and medical humanities - in order to critically reflect on the underpinning assumptions of research on this topic and to identify any new areas of investigation. The questions and prompts within the interview therefore probe participants’ voice-hearing experiences from a wide variety of angles, reflecting an array of different explanatory concerns. For example, a linguist added prompts to explore the tone of the voice, the intentions “behind” what is being said, and whether the voice expresses itself non-literally (leading on from therapeutic approaches which focus on the specific content of voice utterances), while a theologian added prompts to further explore the spiritual dimensions of participants’ experiences, given how common spiritual explanations for voices can be among clinical and non-clinical voice-hearers (Cook, 2018; Peters et al., 2016). The interview was further refined through consultation with clinicians and piloted with a service-user with lived experience of voices. The resulting interview schedule therefore widens the focus on voice experience, potentially enabling connections to be drawn between insights and approaches from a range of disciplines with established areas of clinical concern.

In general, our approach followed phenomenological principles including beginning with general (and avoiding leading) questions, avoiding being prescriptive about what was to count as a ‘voice’, and being careful to distinguish voices from thoughts and other external voices (as in Woods et al., 2015). To minimise any theoretically-driven presuppositions about which aspects of the voice-hearing experience were to be considered important, we avoided asking about voice character, personality and presence until the latter part of the interview.

**Introductory comments**

“For this interview I’m going to be asking some questions about the different kinds of experiences you’ve been having recently. Quite a few of the questions are going to be about hearing voices that other people cannot hear. People sometimes worry about talking about this topic: Do you have any concerns? Is it OK to ask some questions about it?”

“If you would like to stop at any point, or would prefer to talk about something else, then it is absolutely fine to do so”

“Some of my questions are going to refer to “voices”, but people can mean lots of different things by that. For some people that might involve sound, while for others it might feel more like someone or something is communicating with you. Others describe their voices as a form of telepathy, or loud thoughts. Our aim is understand these experiences in terms that make sense for you, so please use whatever language you’re most comfortable with.”

**i. Initial voice description**

“Could you try to describe to me some of the voice (or voice-like) experiences you’ve been having?”

Prompts:

- *How, if at all, are these experiences different from your own thoughts?*
- *How, if at all, are these experiences different from hearing the voice of someone who is present in the room?*
- *Are there any other senses (e.g. images, tastes or smells) involved in the experience?*
- *Does it have a location (i.e. does it feel like you can tell where the voice is coming from)?*
- *Number of different voices/identities (if volunteered)*
- *If there is more than one voice, how do you distinguish between them? i.e. do they sound different, do they have different characters, do they say different things?*
- *When was the last time you had this kind of experience?*

**ii. General background**

“Could you tell us a bit about what life was like for you, and how you were feeling, when you first started having these experiences?”

Prompts:

- *Do you remember when you first heard a voice? (establish age estimate)*
- *If so, can you describe it?*
- *Was it similar to your recent voice/voice-like experiences?*
- *How were things going at the time?*
- *When did you first seek help or treatment?*

**iii. Emotions, feelings & anticipation**

“How does it feel when you have the experience?”

Prompts:

- *What kinds of moods or emotions are associated with your voices?*
- *Do you know when you are about to experience a voice? If so, how?*
- *Does your body feel different when you experience voices?*
- *Are there particular times or places when the experience is likely to occur?*
- *How do you feel towards your voice? Is this always the case, or does it vary?*

**iv. Communication & content**

“Do your experiences contain messages of any kind?”

Prompts:

- *What kinds of things does the voice say?*
- *Are there specific words or sentences that are used? Can you give me any examples?*
- *Do you always understand the message (what is being said)?*
- *How is it expressed? (e.g. friendly, unfriendly, angry, dominant, commanding, supportive, loud, quiet)*
- *How can you tell how it is expressed? Is it the tone of voice, for example?*
- *Is the message ever spiritual or religious?*

**v. Character & agency**

“*Does it feel as though the experiences have their own character or personality?*

Prompts:

- *Do you know who they are? If so, how?*
- *Do they remind you of anyone you know or have known?*
- *If so, is it the voice or what they say that reminds you of this person?*
- *Are they always in the same form (e.g. a voice, a vision, a presence?)*
- *Do they mean well?*
- *Do they know things that you do not?*
- *Are they a spiritual being?*

**vi. Change over time & life impact**

“Have your experiences changed at all since they first started?”

**Prompts**

- *Has it tended to happen more or less over time?*
- *Over time, have you been able to influence your voices? If so, how?*
- *How, if at all, have your voices affected your relations with other people?*

**vii. Personal meaning & interpretation**

“Why do you think these experiences are happening?”

Prompts:

- *What do the voices mean to you?*
- *Do you consider hearing voices to be a special ability or skill?*
- *Have they been caused by something that has happened to you, do you think?*
- *How do you think other people view this experience?*
- *Have you developed any ideas about why you have these experiences?*
- *Are you a religious or spiritual person?*
- *Have any of your family members had similar experiences?*

**viii. Other experiences**

“Is there anything else we haven’t talked about yet, but is an important part of your experience?”

Prompts:

- *Presence of any other senses or multisensory elements (if not already covered)*
- *Have you also had any other experiences that you think might be linked?*

**Section 2. Codes used in qualitative analysis.**

| Code | Definition | Example |
| --- | --- | --- |
| **Sensory Qualities and Modality** | | |
| Auditory | Experiences with an auditory quality directly comparable to everyday speech. Can be perceived as coming from inside or outside the head. | It’s there all the time and it’s a voice, I hear it as clear as days like you speaking to me [Mike]  They’re stood behind us talking directly like into me ear kind of thing…well not whispering to us but like very close [Kath] |
| Thought-like | Experiences described as being similar to, confused with or indistinguishable from the voice-hearer’s own thoughts. | It’s trying to find whether that’s my thoughts or whether that’s actually the voices [Alex]  Difficult to differentiate between what is real and what isn’t and…what are my thoughts and…then… I don’t know how much I have control over these kind of things which come into my head. [Gail] |
| Non-verbal | Experiences of hearing non-verbal voices (for example moans, screams, laughter) or sounds such as bangs, clicks or music. | It could just be like…an actual bark or like…a shout or a noise, you know like someone that is more just ehm like a bang on the table or something like that [Bill]  I could hear generally just moaning noises, sexual noises, like long, deep breaths, stuff like that [Fred]  I hear as well like regular like screaming and … and kind of … kind of maybe like you’d say evil laughing, so they’re not even saying anything, or like someone crying, screaming and wailing. [Olivia] |
| Visual hallucination | Experiences of seeing things which others do not. Specifically, references to seeing things “out there” “in the world” and not simply “in the mind’s eye.” | When I’m sat in me mam’s, she’s got like a window over there, and you can see right, you can see right down into the cemetery, cemetery wall, I can see people standing in it…They’re just like tall shadows and then … I’ll be sat and something will run over, and I know there’s nothing there, then there’ll be things scuttling across the floor and there’s nothing there. [Kate]  I was in school, and I was sat in just a science lab, and I heard my granddad, who had just died, talking to me, eh, and I saw his body on the table. [Dan] |
| Visual imagery | Experiences of “seeing things in my mind’s eye” or intrusive visual imagery. Specifically, references to seeing things “inside the head” or that are clearly distinguished from an external visual perception. | And in like me mind’s eye, like I kinda glazed over, and in me mind’s eye, I kinda had a full on vision of me grannie and just like coming through with loads of light and sat in a wheelchair. [Leah] |
| Tactile hallucinations | Experiences of being touched by something or someone who is not otherwise perceived. Distinct from “bodily experiences” (see below). | I mean I’ve had the feeling of feeling like people poking you or like shoving you and things like that. And then …Being touched … when no one’s touching you … I feel people sitting on the bed, I can feel someone putting their hand on my leg, I can feel … I can feel brushes up against my arms that aren’t there. [Olivia]  Sometimes I have felt like something touch me shoulder, and I’ve felt like, like flipping out…[Iris] |
| Olfactory hallucinations | Experiences of smelling things which others do not. | I sometimes smell smoke, like there’s been an explosion or a car crash. [Dan]  Me mam’s other sister, she died and there was a particular type of smell in her house when she was dying, and it was, it was like a proper sweet, sickly smell, but I’ve smelt that when I’ve gone in me mam’s before and I say what the bloody hell have you been spraying now, that stinks, she says, I haven’t sprayed anything. [Kate] |
| Gustatory hallucinations | Experiences of tasting things which others do not. | [Not allocated] |
| Dissociation | Experiences of being detached from reality, feeling like one’s body or surroundings are in some way unreal, being inhabited by voices, or of experiencing loss of memory or consciousness. | And then I’ve, or I’ve found myself in a completely different place than where I was say half an hour ago, so I know I’ve moved and I just don’t remember. [Dan]  I don’t remember what I did say, what I said, because it wasn’t me talking. [Neil] |
| Bodily states | Experiences of changed bodily states while hearing voices, for example elevated heart rate, feelings of being physically controlled by others, feelings of warmth throughout the body, unusual interoceptive awareness, localized pain. Distinct from “tactile hallucinations” (see above). | Headaches, really bad headaches … as soon as I started to get upset, so as it was starting to build up, it would start with a headache…I’m feeling as if the head’s in a vice. [Jade]  Yeah, it doesn’t feel like it’s my body, it feels like it’s theirs and like oh well they’ve took over it! [Will] |
| Felt presence | Experiences of feeling someone or something is present even when it cannot be seen or heard. | It has no figure and no shape and no form … you don’t know what it is yet, so you can only feel the experience as a presence, you know? [Bill]  Yeah, it feels like they’re with me, it’s, I just feel the presence of someone with me all the time, watching me [Liam] |
| Multimodal voices | Experiencing a voice in more than one sensory modality; that is, a hallucination in a non-auditory modality attributed to the same identity as the voice. Visual, tactile or olfactory perception of the voice may be concurrent with hearing it or occur at a different time point. | If I’m sitting alone in a room, I can her directly talking to me as if she was in the room with me, because I see her as I hear her [Orla]  I also see them…But they’re actually not full people, like form, like there’s a hole in their face and stuff, so I don’t actually see the picture, I just see them [Violet] |
| **Spatiality** | | |
| Internally located | Experiencing the voice as coming from inside the head or body. | It sounds … as though there’s literally a person inside of my head, speaking. [Ryan] |
| Externally located | Experiencing the voice as coming from outside the head or body. | It’s when I’m feeling really anxious, I’ll have like this dark, deep, bellowing voice, what I never, I’ve never heard, like outside of the head. [Fran] |
| Egocentric | Experiencing the voice as emanating from a position relative to the voice-hearer (for example, “next to my ear”). | It only happens on the right side of my head that I can her them more…Yeah, like … maybe at like two feet away. [Xander]  Like a whispering that sounds either really close to me ear [Page] |
| Allocentric | Experiencing the voice as emanating from specific locations in the voice-hearer’s surroundings. | 99% of the time it was voices that I heard, sometimes I heard footsteps, but it was all me witnessing things outside me house. [Jade] |
| Boundary voices | Experiencing the voice at the borders of perception (“just out of earshot”) or at spatial thresholds (coming from just outside the room or house, through the walls, “round the corner”). | I could hear them saying things and that through the wall there [Carl]  Just through the wall, aye, through the wall, and like in a separate room. [Fred] |
| **Control and change** | | |
| Non-volitional Occurrence | Voice-hearing experiences are described explicitly as being outside the voice-hearer’s control. | No, no, like I say, I’ve got no control…She’s just there when she’s there. [Dawn]  It’s not something you can control…Whatever’s being said, it’s … it’s outside of your own conscious power, that’s as best as I can describe. [Bill] |
| Volitional  Occurrence | Voice-hearing experiences can be brought on intentionally, all or most of the time. | [*Interviewer:* *Do you feel like you have control over that situation? Can you, could you stop it if you wanted to, like when you’re in public*?] Yes, ah ah, yeah. [Jane] |
| Ability to influence voice | The voice-hearer is able to change the attitude, behavior or content of their voices. The change does not necessarily have to be lasting, achieved through conversation, or felt to be beneficial. | I’ll have a big conversation back…So sometimes they’ll ease it off, sometimes it allows us to manage it better…Otherwise it comes back a bit more intense and makes the situation slightly worser. [Alex]  Ehm …sometimes I talk back… Yeah, I try to stop them from talking back at us… It’s getting less happening now, so … it’s … getting … easier to block it … [Matt] |
| Change in influence | The voice-hearer’s ability to influence their voices (see above) has changed over time. | Like I said, it depends like if I’m in like a normal state, then I seem to have a bit more control over it, over what I can like influence and stuff like that…But when it goes up to the other end of the scales, it just feels like they’ve got the control of me…[Will]  For the past couple of weeks, yeah…Ehm, they’re still there, obviously, they are still there, but I have been trying to control them. [Violet] |
| Change in frequency | The frequency of voices (how many times a day/week voices are heard) has changed over time. | A lot more often, it’s nearly every day of the week…It used to be just once or twice a week when I was younger [Orla]  But, and then finally I got, he got us on medication to like try us out, and ehm, it was, it’s working. So, so I don’t hear the voices as much…But I did hear them all the time, and I didn’t know why. [Violet] |
| Change in number or structure of voices over time | The number of different identifiable voices, or their physical properties (for example, loudness) have changed over time. | In all honesty, ehm, the other voices, like I say, it just feels kind of like they just kind of cropped up one day. [Anthony]  It’s not constant, it’s not seven voices at once anymore, it’s just like maybes the odd one or two or something like that. [Leah] |
| **Affect and content** | | |
| Voices elicit positive emotions | Voice elicits positive emotions. | I’ve got some positive voices as well. I’ve got some voices, there’s one voice and she, she likes to just talk.  Sometimes she just talks about what’s going on, like she’ll comment on a tree or a bush or like what the sky is like. [Nina]  Like she makes us feel really good. So like where … when she’s there, like I go out and I feel glorious and I feel like I can do what I want, but she’s not there very often. [Kate] |
| Voices elicit negative emotions | Voice elicits negative emotions. | Angry, he just winds me up he does, that’s all he does is wind me up, just constant [Mike]  But it’s mainly just distress and all the feelings that come with distress, do you know what I mean? So you can be frustrated that you’re distressed, you can become like hysterically sad…[Olivia] |
| i) Anxiety-associated | Voice prompts specific feelings of anxiety or worry, or is elicited or exacerbated by anxious states | So sometimes the voices would say, ‘who’s that in the street?’ Or, ‘what’s that car doing?’ And they’d start to get panicked, thinking that somebody was coming to hurt me, and that would raise my anxiety. [Jade]  Well if, well if I hear, like yesterday I heard it, it made us jump and look around, and then obviously I listened and I thought, just go away, leave us alone [Zara] |
| ii) Depression-associated | Voice prompts specific feelings of sadness, despair or depression, or is elicited or exacerbated by depressive states | It … like I said, it feels cripp … it cripples you, it’s a crippling sadness. Like … I’ve … I’ve said for such a lo … a long time, I’d love just a day, just one day of no voices, no mania episodes, no depressive episodes, no … self-harm, no suicidal thoughts, no euphoric illusions, just one day [Olivia]  It just draws down … anger, sadness … all really negative things. [Harry] |
| iii) Fear-associated | Voice prompts specific feelings of fear or dread, or is elicited or exacerbated by fearful states | It’s, is it something that, it scares you every time, when you realise that there’s nobody there, it’s a scary thought to be left with. [Bill]  I have a genuine fear that they’ll kill me one day and people won’t know that it was … them taking control over me. [Olivia] |
| iv) Paranoia-associated | Voice prompts specific feelings of paranoia or distrust, or is elicited or exacerbated by paranoid states | And so whenever I get really paranoid, they overlap a lot, and it’s a lot of like, I can hear whispering and stuff ehm [Dan]  When I’m hearing through a wall, like listening through the wall, that’s more like the paranoia, and I, and then I tend to listen for, what I, what I thinks they’re saying anything [Fred] |
| Simple linguistic structure | Voice predominantly or exclusively uses single words or simple phrases. | When it comes to the voices, it’s ehm, I don’t actually get full sentences or words, it’s like a mumble or a whisper [Chris]  There are sometimes … times through the day where I can hear my name mentioned. [Bill] |
| Directly address voice-hearer | Voice directly addresses the voice-hearer (using the second person address). | I can tune out, and then the other voice will aim a lot at me. ‘Why are you talking to him?’ ‘Why are you listening to him?’ ‘Why are you trying to make sense of him?’ [Ryan]  I was in me flat, and it was threatening me, saying she wanted me out the flat by half four that day. And then obviously … I telled me dad and stuff, and then it was, she was just calling us a brass and stuff …and it’s like threats all the time. [Emma] |
| Voices comment on voice-hearer | Voice comments on the voice-hearer’s current actions or thoughts. | Just the same thing over again, over and over again, sort of ‘you’re horrible’, ‘you’re disgusting’, sort of thing. [Dan]  Yes, certainly, just contradicting or commenting on things I’ve been doing around the house, as I’m doing them, as if someone’s actually there, watching you at the time they’re commenting, you know [Alex] |
| Voices converse with voice-hearer | Voice-hearer can have a conversation or dialogue with the voice. This does not include answering back to voices without meaningful reply; voices must be able to converse and respond flexibly to voice-hearer. | Sometimes like, depending on what mood I’m in, I can … give as good back, and like if I hear her whispering, I’ll answer back, do you know what I mean, and I’ll give her digs back [Emma]  Well when she was then, it was just like random words and things that I would hear in my head, where now it’s more like a conversation. […] you can have a full-on conversation, like back and forth like she was a separate person, yeah, ah ah. [Jane] |
| Commanding voices | Voices commands or instructs the voice-hearer to do things, whether harmful to self or others, or benign/helpful. | Eh, I was like hiding in the bushes across the road, because eh, it was, like there was, the voices in me head were telling us, you’ve got to stay, stay out here and try and catch them. [Fred]  Just “stab people”. [Matt]  Yes, they help or tell me what to do [Eric] |
| i) Voice-hearer follows commands | The voice-hearer carries out actions when instructed by the voice. | Sometimes I do like ehm … listen to Mark and self-harm [Violet]  And then they told us to go to the rail station and to jump train. Ehm, so I went there a few times, and I’d been thinking to meself, I cannot get on this train, I haven’t got any money, you know, I can’t get on this train. And then they said, if you don’t do it, you’re going to die, you’re going to get stabbed, you’re going to get knifed. So I jumped on the train and went. [Leah] |
| Abusive/  violent voices | Voice says things which are hostile and may threaten or insult the voice-hearer. | …Sometimes it can be what I feel maybe more internal, where it could be one or more personalities, but it is always aggressive. [Bill]  …Despondent male voice who’s be like, ‘no you are absolutely worthless’. [Page] |
| Positive/  helpful voices | Voice says things which are friendly, helpful or positive. | …The times when I’ve been able to pinpoint those to like the woman, ehm, when I’ve got my kids and things, I can find that sort of soothing at times…. it’s very attentive towards kind of my, my kids. [Anthony]  …I don’t know, sometimes she can be OK and seem like a nice person. [Orla] |
| Companionship from voice | Voice provides companionship. | …The green lady who I used to, when I was a teenager, I used to go out, ehm, when I needed to get out the house, from my parents, my family and so on, I would walk and talk to her, and she’d be the one that always held my hand. [Eric]  Yeah, like I’ve become quite attached to them…And even the bad things that they’re doing, saying stuff now, sometimes if I’m sat there and I don’t, I don’t hear anything for half a day or something like that, or twenty four hours, I start looking for them.…Because I’m so used to hearing them now, they’re like part of us, almost keep us company.[Leah] |
| Voice knows more than voice-hearer | The voice is experienced as possessing knowledge (about events, other people, the future) that the voice-hearer does not. | …They’re both very certain, like they know, like there’s something telling them, wherever they’re from. Ehm, so yeah, they do, I think they do, they know something that I don’t. [Ryan]  She’s like, she’s from England, she just won’t tell me where [Xander] |
| **Agency and Character** | | |
| Recurring voices | The same voice is heard on multiple occasions. | Yeah, yeah, it tends to be there, yeah…The same voice, yeah. [Ian]  So I suppose over the last couple of months I’ve sort of been hearing I think up to seven different voices. [Dan] |
| Voices recognisable from voice-hearer’s life | The voice heard is the voice of a person in the voice-hearer’s life. | Ehm, well they’re people that I know or have seen, ehm … and they’re linked to me past. [Hugh]  …All of a sudden it turns out to be the voice of somebody that you might have had an altercation with or somebody that you’ve never gotten on with in life. [Bill] |
| Change in character or personality of voices across time | The “personality” or psychological characteristics of the voice change in ways which are sustained over time. | Ehm, I feel like he gets angrier day by day. He always seems to be coming up with new ways to like try and make like, manipulate me or ... try and get me to do things. [Xander]  It started off with the sex or drugs ring, then it became undercover police involved in that, then it became the arrests of the prostitutes, then it became the arrests of the people, the perpetrators, or the people who were organising it. Then there was a scenario of the bad men looking for eh … the snitch. And then it became the policemen initially looking at me, thinking that I had been telling the criminals what, you know, where the people were and the rest of it, and now it’s them surveying. So it’s (laughs) it’s been a, it’s been a sort of like a scenario that’s working its way through. [Jade] |
| Absent agency^§^ | An auditory experience in which there is little or no perceived communicative intention. | I get clicks in this ear, and I get clicks in this ear…And I find that, that … I don’t know whether it’s the voices that are coming through that are doing it, or whether I’ve programmed meself to do this [Leah] |
| Agency without individuation^§^ | An auditory experience in which agency is represented but without a distinguishable agent; directed towards the voice-hearer. | Just things like that, name being called, sometimes laughter, that’s other people can’t hear. [Bill]  But it’s like they’re having a conversation together about me, criticising on everything I do….It’s like a little gang…two or three of them’s the gang leader….the ones that getting, like basically give instructions to the other ones. [Toby] |
| Internally individualised agency^§^ | An auditory experience identifiable as consistently emanating from a specific agent whose identity is not known to the voice-hearer. | I’d say with the other voices you can tell because some are sort of what you’d consider a stereotypical deep male voice…Whereas another might a higher pitched, stereotypical woman’s voice. [Dan]  It’s a female voice, as if she’s in the room with me, ehm, and… I wouldn’t say she’s nasty, she’s just really stern and to the point [Dawn] |
| Externally individualised agency^§^ | An auditory experience identifiable as consistently emanating from a specific agent whose identity is known to the voice-hearer. | It was me nanna’s voice telling us not to do silly things…I’d just hear her voice, but it was like how it is now, it was echoing around us. [Zara]  So I didn’t realise I had it until I moved, because when I was up there, there was a lad who hadn’t, who had mental health problems and he couldn’t stop saying that derogatory word over and over and over….So that’s went into my head, and when I’ve come down here it hasn’t stopped, I’ve kept on hearing the same voices. [Sean] |
| Minimal personification* | The voice has few person-like qualities; is attributed to a person or described as being “like a person” but without further elaboration. Person-like characteristics tend to remain stable over time and follow a single theme (e.g. the voice is “mean”, or a “nasty man”). | The most recognisable voice to me isn’t of anybody that I’ve ever met before, sometimes it doesn’t even sound human. In its barest form ... a bark that speaks English. [Bill]  …It’s usually a man’s voice that’s more negative for me. Most of the time, like I say though, it just sounds like chattering, all the time. [Anthony] |
| Complex personification* | The voice is described as having more than one kind of person-like quality. These may include elaborate descriptions of intentional states (the voice wants/thinks/feels), agency (the voice will “make something happen”), or identity (the voice “comes” from somewhere or has a specific and idiosyncratic ontological status). Complexity is not a simple function of the frequency, quantity or topic of speech, but will typically involve a voice being attributed multiple, qualitatively different person-like qualities (e.g. voice has an identity *and* multiple mental states) which may vary over time. | Like Bex will be happy and you can tell she’s happy, or you know I wouldn’t say she’s sad but you can tell when she’s not happy but she won’t be sad. [Xander]    I describe her as the like pernicious one… if I feel down, she’s the one that would sort of be in the mirror, ehm, sort of hovering there, saying, ‘you need to, you’re a shit human being, you need to lose weight’ or something like that, if I’m feeling quite negative anyway. But she’s also the one that… was telling me to go in front of traffic because I couldn’t go because I was having a panic attack about work, and she was the one that just said, ‘if you go, just step, all you need to is just step, you’ll break a leg, you’ll be in hospital, you’ll be safe, I’ll keep you safe’.…she can go a bit too far sometimes [Eric] |
| Archetypal features | The voices conforms to a recognised cultural, mythical or religious stereotype that recurs in common stories, folk tales or spiritual texts. | Ehm, it’s a very, it’s not a voice I would recognise, it’s no one in my past or anything like that, it’s just a very kinda ... I want to say almost demonic really in the way that it talks. [Anthony]  He’s very like, sort of quite clean-shaven, very clean-cut, he just looks like a, he’s sort of very monotone, and it’s always something about, something bad that’s going to happen that you need to prepare for. And it, I hate to say this, but it’s sort of like he’s like an official of some sort…He sounds very like eh, commanding, and he sounds very sort of like, you know RP accent. [Dan] |
| **Social context and interpretation** | | |
| Voices important to identity/sense of self | The voice is an important part of the voice-hearer’s identity. If gone, the voice-hearer may feel like they were missing a part of themselves. | I ... there’s times, when I, if I’m having an episode, and I can thoroughly believe that I’m being spoken to by archangels and they’re telling me to do something, they’re giving us a job to do. [Leah] |
| Positive impact on relationships | The voice has impacted positively on the voice-hearer’s relationships. | But it’s had a positive rela ... eh effect with some of my relationships with my friends, but if anything, it’s sort of made me like closer to them. [Dan] |
| Negative impact on relationships | The voice has impacted negatively on the voice-hearer’s relationships. | I’d say it’s definitely had a negative impact in a lot of ways because people a lot of the time struggle to understand, and they don’t really know what’s going on, or they think it’s, you know, sometimes people think you’re like faking it [Dan]  I went out with one of me mates, and I’ll see one of them, and even if their lips, even if they’re just breathing... I might think that they’re talking about us, and then, and that might trigger like some bad thoughts, some different thought patterns. [Brad] |
| Self-stigma regarding voices | The voice-hearer applies to themselves negative stereotypes about hearing voices. | Then I hate myself because I feel like I’ve brought them there. (pause) Because to me like … obviously with the threats, they’re in danger, and I’ve brought them there. [Emma]  Just makes us feel like I’m weak, you know, weak-minded by getting it, a bit worthless. [Sean] |
| Suicidal thoughts or actions | The voice-hearer has attempted suicide or reports suicidal ideation. | They’re very bad, it sort of came to the point where I was going to overdose again. [Orla]  And that was when at me highest point of self-harming, taking overdoses and that. [Zara] |
| Sleep disruption | The voice-hearer reports insomnia, unusual sleep patterns or other problems with sleep. | So there was a second night where I didn’t sleep. Ehm, and that was when I decided, I need help. [Jade]  Yeah. Like it distrup … distrup (laughs) … I can’t even say the word! It disrupts like sleeping and everything. Like I dread going to bed. [Emma] |
| Traumatic context around onset | The voice-hearer reports particularly difficult, stressful or traumatic events in the months prior to their first voice-hearing experiences (whether or not they identify a causal relation). | Ehm, it was very, it was very, very, very … it was the hardest time of my life…When I had these. I’d … me mother had just had cancer….Me husband left us when she got the cancer. [Leah]  Well I was made redundant from a job…which left us homeless. I’d already experienced like periods of homelessness in my life, but I’d always managed to get into hostels. [Bill] |
| Trauma interpretation | The voice-hearer attributes the voices at least in part to past severe or adverse events, including: emotional, physical, or sexual abuse; witnessing or experiencing violence; serious accidents or injuries; combat experience, sustained bullying; sudden bereavement. | The little boy was, I’m assuming it’s a boy, little baby that I had when I was fourteen year old, I had to have a termination at twenty two weeks, and it was little boy saying, ‘I still love you’…I knew it was Daniel, because I called him Daniel, ehm … obviously I’m not going to get meself upset, it’s just … Daniel and me nanna’s voices were very reassuring, knowing that I was at peace because I never got over that termination. [Zara]  So I think that ehm, like I was bullied at school, I was like bullied at like home with you know kids on the street and things like that. [Xander] |
| Biophysical interpretation | The voice-hearer attributes the voices at least in part to biological causes, including: something going on with the brain; physical health issues; sleep disruption; illness; impact of drugs and alcohol. | It was really bad on the legal highs and the Spice…That’s what it was. [Toby]  I think I entered into a total state of eh, I don’t know, psychosis. [Brad] |
| Stress interpretation | The voice-hearer attributes the voices at least in part to a stressful event or period of sustained stress reflecting mild or moderate adverse life events, including changes in education, employment or housing; turbulent family relationships; bereavement. | Ehm, I had a couple of stressful things going on at the time. [Ian]  It depends on how eh settled I feel. If I start to feel a bit nervous or a bit anxious….then the voices come a bit closer. [Jade] |
| Idiosyncratic interpretation | The voice-hearer has an idiosyncratic causal explanation for their voices, for example that they are implanted by foreign agents. | I think they’re solely a punishment, they weren’t around before I deserved punishment and now they are, and then once I’m gone, they’ll be gone. [Ryan]  And they started … the beginning of November last year, when I believed I was witnessing ehm, either a … a sex ring or a drug ring outside…Ehm, and initially it was only a couple of nights a week, gradually it became more and more, ehm, and what I believed I was hearing was eh … turned into an undercover operation, involving police officers. Who were part of, who had infiltrated the ring [Jade] |
| Supernatural/spiritual interpretation | The voice-hearer attributes the voices at least in part to spiritual or supernatural causes. | Sometimes I try and put something to it, so sometimes I think, oh I think it’s a ghost and sometimes I think it’s like … worse than a ghost(!) and wind meself up! [Page]  I think I’m being told to change me life…I think I’ve been on the wrong path, and I think I’ve been, I’m being placed on a different path…I was spiritual, but I wasn’t big into God or Jesus or anything like that, but I am now, like, definitely. [Leah] |
| Family narrative | The voice-hearer describes voices as being “in the family,” linking their experiences to the experiences of family members. | Me nanna now that I hear, she had a psychotic breakdown and she went to kill me Auntie [name], this is me nanna’s sister but we used to call her Auntie [name] and she was sectioned ten year prior to her dying. [Zara]  I don’t, but there is, me mam thinks it is sommat wrong with me real dad, because like he’s very recluse, like he keeps hissel to hissel, doesn’t go out the house, eh, and he’s been like that for years, and he doesn’t have nothing to do with her anymore. So me mam thinks there’s always been something wrong with me dad, but now, now this has happened to me, she thinks that could have come from me dad, she thinks it might have been passed down. [Fred] |

* denotes codes that could only be used exclusive of one another.

^§^ Codes for agency derived from Wilkinson & Bell (2016)

**Section 3. Table S1. Comparing odds ratios for all codes against complex personification by section and rank ordered.**

| **Sensory qualities & modality** | **Log OR** | **Low CI (2.5%)** | **High CI (97.5%)** |
| --- | --- | --- | --- |
| Visual hallucinations | 2.20 | 0.01 | 4.38 |
| Visual imagery | 2.13 | -0.97 | 5.24 |
| Multisensory | 1.36 | -0.10 | 2.82 |
| Dissociative | 1.08 | -0.31 | 2.48 |
| Olfactory | 0.89 | -0.43 | 2.20 |
| Bodily states | 0.76 | -0.63 | 2.15 |
| Non-verbal | 0.69 | -0.60 | 1.99 |
| Auditory | 0.31 | -2.18 | 2.80 |
| Thought-like | 0.25 | -1.02 | 1.52 |
| Tactile halls | 0.24 | -1.26 | 1.74 |
| Felt presence | -0.17 | -1.43 | 1.10 |
| Gustatory* | **-** | - | - |
| **Spatiality** |  |  |  |
| Internal | 0.45 | -0.88 | 1.78 |
| External | 0.13 | -1.46 | 1.73 |
| Egocentric voices | 0.00 | -1.31 | 1.31 |
| Allocentric voices | -0.10 | -1.47 | 1.28 |
| "Boundary" voices | -0.28 | -1.62 | 1.06 |
| **Onset & change** |  |  |  |
| Volitional | 1.56 | -1.71 | 4.82 |
| Change in influence | 0.93 | -0.98 | 2.85 |
| Change in frequency | -0.10 | -1.47 | 1.28 |
| Ability to influence | -0.21 | -1.64 | 1.22 |
| Structured longitudinal change | -0.51 | -2.07 | 1.05 |
| Non-volitional* | - | - | - |

* codes at 0% or 100% prevalence not calculated.

| **Affect & content** | **logOR** | **Low CI (2.5%)** | **High CI (97.5%)** |
| --- | --- | --- | --- |
| Positive and helpful | 3.89 | 1.98 | 5.80 |
| Voices elicit positive emotions | 3.50 | 1.66 | 5.33 |
| Companionship | 3.19 | 1.39 | 4.98 |
| Conversational | 2.56 | 1.01 | 4.12 |
| Fear | 1.10 | -0.29 | 2.48 |
| Follow commands | 0.85 | -0.51 | 2.20 |
| Voice knowledge | 0.76 | -0.53 | 2.05 |
| Depression | 0.68 | -0.61 | 1.97 |
| Direct address | 0.61 | -1.17 | 2.39 |
| Commenting | 0.34 | -0.94 | 1.61 |
| Anxiety | 0.28 | -1.06 | 1.62 |
| Commanding | 0.10 | -1.26 | 1.45 |
| Paranoia | 0.00 | -1.27 | 1.27 |
| Abusive violent | 0.00 | -1.91 | 1.91 |
| Suicidality | 0.00 | -1.27 | 1.27 |
| Simple structure | -1.10 | -2.48 | 0.29 |
| Voices elicit negative emotions* | - | - | - |
| **Agency & character** |  |  |  |
| Absent agency | 2.35 | 0.08 | 4.61 |
| Recurring | 1.68 | -1.35 | 4.71 |
| Character change | 1.61 | -0.18 | 3.40 |
| Archetypal | 1.40 | 0.06 | 2.74 |
| Externally individualised | 0.85 | -0.45 | 2.14 |
| Agency without individuation | 0.76 | -0.53 | 2.05 |
| Recognisable | 0.59 | -0.69 | 1.87 |
| Internally individualised | 0.00 | -1.46 | 1.46 |
| **Social context & interpretation** |  |  |  |
| Family narrative | 1.44 | -0.14 | 3.01 |
| Voices Important to identity | 0.93 | -0.98 | 2.85 |
| Idiosyncratic narrative | 0.85 | -0.51 | 2.20 |
| Supernatural narrative | 0.51 | -1.05 | 2.07 |
| Stress narrative | 0.44 | -0.86 | 1.74 |
| Positive impact on relationships | 0.43 | -2.42 | 3.27 |
| Self stigma | 0.17 | -1.10 | 1.43 |
| Biophysical narrative | 0.00 | -1.46 | 1.46 |
| Traumatic context around onset | -0.64 | -1.96 | 0.69 |
| Trauma narrative | 0.00 | -1.46 | 1.46 |
| Negative impact on relationships | -0.24 | -1.74 | 1.26 |
| Sleep disruption | -0.44 | -1.74 | 0.86 |

* codes at 0% or 100% prevalence not calculated.

**Section 4 Table S2. Strongest 10 associations among all codes with commanding voices and trauma around voice onset**

| **Commanding voices** | **Log OR** | **Low CI (2.5%)** | **High CI (97.5%)** |
| --- | --- | --- | --- |
| Suicidality | 3.35 | 1.15 | 5.55 |
| Follow commands | 3.22 | 0.31 | 6.14 |
| Multisensory | 2.93 | 0.01 | 5.86 |
| Recurring | 2.91 | -0.14 | 5.96 |
| Abusive/violent | 2.45 | 0.13 | 4.77 |
| Direct address | 2.06 | 0.23 | 3.88 |
| Trauma around voice onset | 1.72 | 0.28 | 3.16 |
| Externally located | 1.61 | -0.03 | 3.25 |
| Auditory | 1.55 | -0.95 | 4.06 |
| Companionship | 1.33 | -0.36 | 3.02 |
|  |  |  |  |
| **Traumatic context around onset** |  |  |  |
| Stress narrative | 1.79 | 0.11 | 3.47 |
| Commanding | 1.72 | 0.28 | 3.16 |
| Externally located | 1.45 | -0.18 | 3.07 |
| Auditory | 1.43 | -1.07 | 3.92 |
| Suicidality | 1.39 | -0.02 | 2.79 |
| Abusive/violent | 1.19 | -0.74 | 3.11 |
| Multisensory | 1.16 | -0.55 | 2.86 |
| Direct address | 1.12 | -0.55 | 2.79 |
| Depression | 1.06 | -0.29 | 2.41 |
| Thought- like | 1.06 | -0.29 | 2.41 |

**Section 5. Felt presence, direct address, and multimodality.**

The “presence” of voices, their linguistic complexity, and their multimodal properties have all been posited as examples of AVH personification (Alderson-Day and Fernyhough, 2016; Deamer and Wilkinson, 2015; Wilkinson and Bell, 2016). We therefore ran a side analysis to further explore their own associations with other aspects of voice phenomenology.

We identified that felt presence was primarily associated with tactile hallucinations (lgOR = 2.41, CI = 0.21–4.60) and fear (lgOR = 1.99, CI = 0.55–3.42). A high odds ratio was also observed between felt presence and voices using direct address (lgOR = 2.22), although confidence intervals for that relationship marginally crossed 0 (CI = -0.007 – 4.45). Multimodal voices were similarly associated with tactile experiences (lgOR = 1.65, CI = 0.06–3.24) but were also associated with depression (lgOR = 2.80, CI = 0.01–5.86). and companionship (lgOR = 2.55, CI = 0.91–4.19), despite their aforementioned association with commands (see main text).

**Section 6. Comparing those with and without complex personification on voice onset & severity scores.**

Table S3 show the average voice onsets and time spent hearing voices for those with and without the complex personification code (note: one participant was unable to be specific about when their voices started). No clear differences were evident for age of onset nor length of time hearing voices.

**Table S3. Age of voice onset and time hearing voices by personification group**

|  | Complex personification | | | |
| --- | --- | --- | --- | --- |
|  | Yes (n = 16) | | No (n = 23) | |
|  | M | SD | M | SD |
| Age of voice onset | 22.31 | 11.25 | 22.94 | 16.50 |
| Months hearing voices | 73.00 | 72.26 | 75.35 | 88.54 |

All participants were assessed using the Psychotic Symptoms Ratings for Auditory Hallucinations and Delusions. For PSYRATS-AH total severity scores were very similar in groups with (M = 25.69, SD = 7.41) and without complex personification (M = 25.83, SD = 5.57, d = 0.02). Overall scores for delusions were generally low (M = 6.825, SD = 7.68), although this was largely due to nearly half the sample (19/40) not reporting any specific delusions in the week prior to interview. When confined to only those with current delusions, there was nevertheless still little difference (d = -0.03) between those with complex personification (M = 13.11, SD = 5.97) and those without (M = 12.92, SD = 5.45). However, the relatively low rates of delusional ideation in the sample suggest that any conclusions about the relations between personification and delusions more broadly be made with caution.

In contrast to the lack of associations with personification, voice onset age was negatively correlated with PSYRATS-AH ratings (Spearman’s *rho* = -0.53) but much less so for delusion ratings (Spearman’s *rho* = 0.14). When PSYRATS-AH ratings were broken down into their physical, emotional and cognitive subscales, this association was driven specifically by emotional items on the PSYRATS (*r* = -0.50); no other prominent correlations were evident (all *r* <0.32). Hearing voices from a younger age, therefore, appeared to be associated with greater emotional distress at the time of the study­ – even if some of those voices started benignly.

**Section 7. References**

Alderson-Day, B., Fernyhough, C., 2016. Auditory verbal hallucinations: Social, but how? J. Conscious. Stud. 23, 163–194.

Cook, C.C.H., 2018. Hearing Voices, Demonic and Divine: Scientific and Theological Perspectives. Routledge.

Deamer, F., Wilkinson, S., 2015. The speaker behind the voice: therapeutic practice from the perspective of pragmatic theory. Psychopathology 817. https://doi.org/10.3389/fpsyg.2015.00817

Fernyhough, C., 2014. The art of medicine: Hearing the voice. The Lancet 384, 1090–1091.

Haddock, G., McCarron, J., Tarrier, N., Faragher, E., 1999. Scales to measure dimensions of hallucinations and delusions: the psychotic symptom rating scales (PSYRATS). Psychol. Med. 29, 879–889.

Parnas, J., Møller, P., Kircher, T., Thalbitzer, J., Jansson, L., Handest, P., Zahavi, D., 2005. EASE: Examination of Anomalous Self-Experience. Psychopathology 38, 236–258. https://doi.org/10.1159/000088441

Peters, E., Ward, T., Jackson, M., Morgan, C., Charalambides, M., McGuire, P., Woodruff, P., Jacobsen, P., Chadwick, P., Garety, P.A., 2016. Clinical, socio-demographic and psychological characteristics in individuals with persistent psychotic experiences with and without a “need for care.” World Psychiatry 15, 41–52. https://doi.org/10.1002/wps.20301

Wilkinson, S., Bell, V., 2016. The representation of agents in auditory verbal hallucinations. Mind Lang. 31, 104–126. https://doi.org/10.1111/mila.12096

Woods, A., Fernyhough, C., 2014. Hearing voices, in: Holden, J., Kieffer, J., Newbigin, J., Wright, S. (Eds.), Where Does It Hurt? The New World of Medical Humanities. Wellcome Trust, pp. 84–85.

Woods, A., Jones, N., Alderson-Day, B., Callard, F., Fernyhough, C., 2015. Experiences of hearing voices: analysis of a novel phenomenological survey. Lancet Psychiatry 2, 323–331. https://doi.org/10.1016/S2215-0366(15)00006-1

Woods, A., Jones, N., Bernini, M., Callard, F., Alderson-Day, B., Badcock, J.C., Bell, V., Cook, C., Csordas, T., Humpston, C., Krueger, J., Larøi, F., McCarthy-Jones, S., Moseley, P., Powell, H., Raballo, A., Smailes, D., Fernyhough, C., 2014. Interdisciplinary approaches to the phenomenology of auditory verbal hallucinations. Schizophr. Bull. 246–254.
